# Light-Stress Influences the Composition of the Murine Gut Microbiome, Memory Function, and Plasma Metabolome

**DOI:** 10.3389/fmolb.2019.00108

**Published:** 2019-10-18

**Authors:** Young-Mo Kim, Antoine M. Snijders, Colin J. Brislawn, Kelly G. Stratton, Erika M. Zink, Sarah J. Fansler, Thomas O. Metz, Jian-Hua Mao, Janet K. Jansson

**Affiliations:** ^1^Biological Sciences Division, Pacific Northwest National Laboratory, Richland, WA, United States; ^2^Biological Systems and Engineering Division, Lawrence Berkeley National Laboratory, Berkeley, CA, United States; ^3^Computing and Analytics Division, Pacific Northwest National Laboratory, Richland, WA, United States

**Keywords:** light stress, sleep cycle, gut microbiome, plasma metabolome, behavior change, memory function

## Abstract

The gut microbiome plays an important role in the mammalian host and when in proper balance helps protect health and prevent disease. Host environmental stress and its influence on the gut microbiome, health, and disease is an emerging area of research. Exposures to unnatural light cycles are becoming increasingly common due to travel and shift work. However, much remains unknown about how these changes influence the microbiome and host health. This information is needed to understand and predict the relationship between the microbiome and host response to altered sleep cycles. In the present study, we exposed three cohorts of mice to different light cycle regimens for 12 consecutive weeks; including continuous light, continuous dark, and a standard light dark regimen consisting of 12 h light followed by 12 h of dark. After exposure, motor and memory behavior, and the composition of the fecal microbiome and plasma metabolome were measured. Memory potential was significantly reduced in mice exposed to continuous light, whereas rotarod performance was minimally affected. The overall composition of the microbiome was relatively constant over time. However, *Bacteroidales Rikenellaceae* was relatively more abundant in mice exposed to continuous dark, while *Bacteroidales S24-7* was relatively more abundant in mice exposed to continuous light. The plasma metabolome after the continuous dark exposure differed from the other exposure conditions. Several plasma metabolites, including glycolic acid, tryptophan, pyruvate, and several unidentified metabolites, were correlated to continuous dark and light exposure conditions. Networking analyses showed that serotonin was positively correlated with three microbial families (*Rikenellaceae, Ruminococcaceae*, and *Turicibacteraceae*), while tryptophan was negatively correlated with abundance of *Bacteroidales S24-7* based on light exposure. This study provides the foundation for future studies into the mechanisms underlying the role of the gut microbiome on the murine host during light-dark stress.

## Introduction

Sleep is a biological process which is essential for life to maintain resiliency of brain and other organs in the body. Good sleep health is characterized by adequate duration, low environmental stressors such as light and noise, appropriate timing, and nutrient uptake resulting in alertness during waking hours (Reynolds et al., 2017). The circadian rhythm, the endogenous biological process that corresponds to external oscillations within the 24 h period in a day, is critical to maintain healthy life. Alterations in the circadian rhythm can cause physiologically unstable states after short-term or long-term shifts away from normal (Mohawk et al., 2012; Abbott et al., 2018). Light exposure is an important factor for mammalian systems to maintain homeostasis and light availability influences their selection of habitat and niche within an ecosystem (Ankel-Simons and Rasmussen, 2008). Short-term alterations in light exposure can cause minor fatigue, whereas long-term alterations can cause sleep-related diseases in both animals and humans (Mukherjee et al., 2015). It is known that light and other types of environmental stress are important factors that influence physical and mental host homeostasis (Moloney et al., 2014; Karl et al., 2018). Not surprisingly, understanding the influences of environmental stress on the host has been an active research field for more than a century (Schwartzman and Ruby, 2016).

The mammalian body is associated with a diverse and tremendous number of microbial cells which are collectively called the microbiome. Specifically, the microbial colonization of the mammalian gut by microorganisms is largely maternally inherited during the birthing process although the settled microbial consortia are constantly influenced by environmental exposures, diet, and direct physical contacts among bacterial species (Koenig et al., 2011; Snijders et al., 2016). The gut microbiome has been shown to play an important role in digestion, disease, growth, host immunity, metabolic homeostasis, and brain development (Tremaroli and Bäckhed, 2012; Gilbert et al., 2018). Of particular interest is the communication between the gut and the brain in response to stress. The gut-brain axis (GBA) has recently been recognized to be key for signaling. However, the contribution of microorganisms living in the gut environment toward signaling via the GBA and how environmental stressors can affect the GBA are still poorly understood (Foster et al., 2017; Thomas et al., 2017). One possible mechanistic link between the gut and the brain are metabolites produced by specific members of the gut microbiome and which can affect host behavior.

Currently, highly advanced molecular biological techniques and analytical instruments including mass spectrometry and NMR have revolutionized our ability to study the effects of environmental stressors on host health and the resulting consequences over time (Gowda and Djukovic, 2014; Markley et al., 2017). In the present study, we investigated whether light stress is associated with functions carried out by the gut microbiome, metabolites they produce and behavior. We exposed three mouse cohorts to altered light schedules for 12 consecutive weeks and analyzed the fecal microbiome following exposure. We then performed motor and memory behavior tests and a metabolomics study of plasma samples. Finally, we evaluated microbiome changes within the altered light-dark exposure patterns, alterations in behavior, and shifts in the plasma metabolome profile due to the light exposure changes.

## Materials and Methods

### Experimental Design

**Figure 1:**
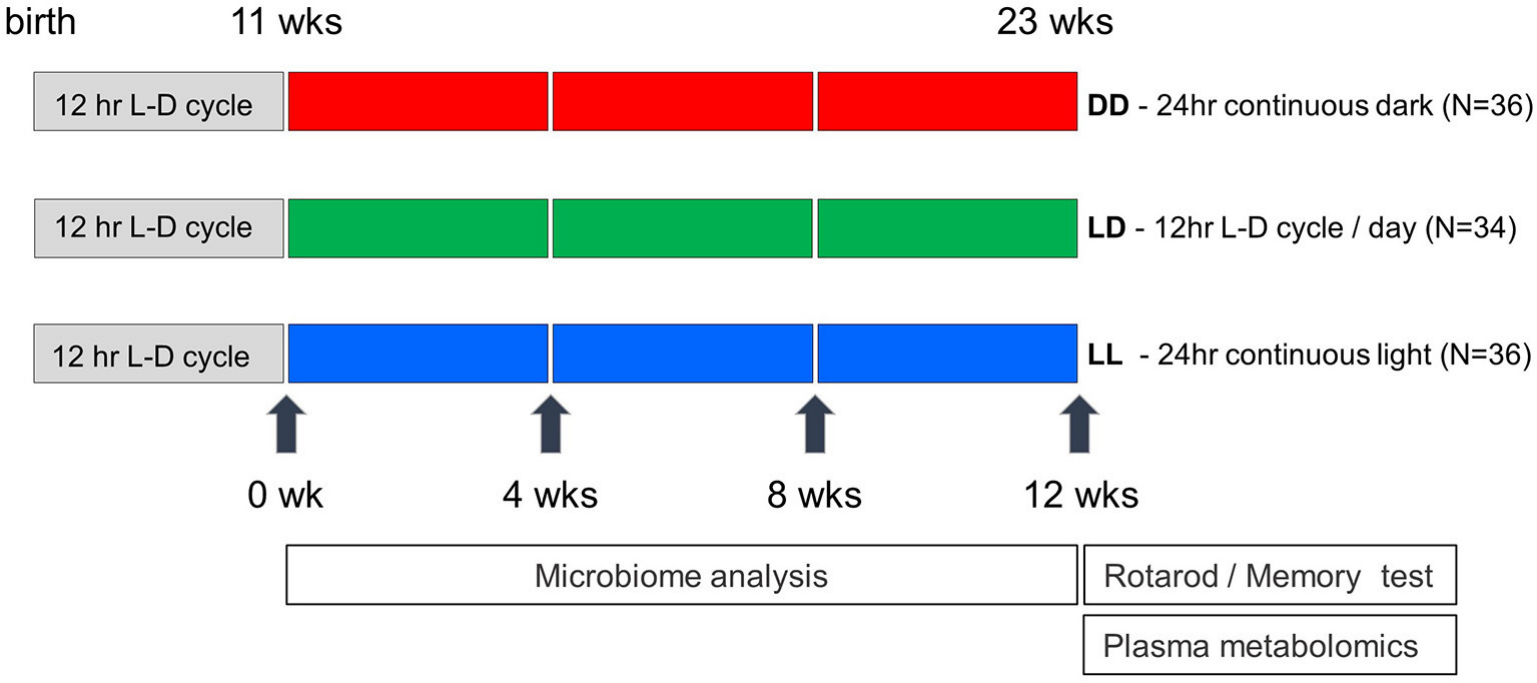
Experimental design of light exposure in the study. Mice were transferred in the light-controlled cages after 11 weeks after birth, and maintained under three different conditions (DD, LD, and LL). Mouse fecal pellets were collected every 4 weeks and were saved until all the collections are finished. Rotarod performance and memory tests were conducted at the week of 23rd, and blood samples were collected a week later.

C57BL/6J male mice (*N* = 106) were obtained from Jackson Laboratories at 5 weeks of age. Mice were group housed until 1 week prior to the start of the light regimen, at which time mice were pair housed. At 11 weeks of age, mice were placed inside ventilated boxes with an automatic light time controller for 12 consecutive weeks. The light and dark conditions were strictly controlled every 12 h and included three different light regimens: Light-Light: LL (continuous light, *N* = 36), Light-Dark: LD (12 h-light and 12 h-Dark, *N* = 34), Dark-Dark: DD (continuous dark, *N* = 36) (Figure 1). Fecal samples from each cage were collected every 4 weeks starting at the beginning of the altered light cycle until week twelve. In total, 96 fecal samples were collected (eight mouse cages for each of three light conditions across four timepoints). At the end of the 12 week light regimen, mice were removed from the light/dark boxes and housed under standard 12 h-light and 12 h-dark cycle light conditions. Twenty four hours after removing mice from the light/dark boxes, two behavioral tests were performed on all mice at 23 weeks of age including the rotarod test to examine motor performance and the passive avoidance test to assess memory potential. All mice were tested during the light phase of a standard 12 h-light and 12 h-dark cycle. Mice were maintained on PicoLab Rodent Diet 20 (5053), housed in standard micro-isolator cages on corn cobb bedding with enrichment consisting of crinkle cut, naturalistic paper strands. Mice had unrestricted access to water and food. Food intake was not measured in this study. One week after the behavioral testing, all mice were euthanized, and blood was collected. Mouse body weights were not significantly different across the three different light regimen at the time of euthanasia (data not shown). Whole blood was centrifuged at 2,500 rpm for 10 min and plasma was collected and snap frozen in liquid nitrogen. The animal use protocol was approved by the Animal Welfare and Research Committee of the Lawrence Berkeley National Laboratory (Protocol File number: 271009).

### Mouse Behavioral Procedures

Mice were tested during the light phase of a standard 12 h-light and 12 h-dark cycle for their ability to maintain themselves upright on a rotating rod. Animals were placed on a spindle at 45 degree angle and subjected to a slow-speed “waiting” mode (4 rpm for 5–10 s) before acceleration. To avoid passive rotation of the mice on the rod we used a spindle with a diameter of 30 mm and a lane dimension of 50 mm. Acceleration was started after the “waiting” period and was set at 20 rpm/min for all mice. Trials where mice fell off in <5 s are likely due to operator error and were repeated and not included in the analysis. Animal falls were detected by a pressure sensitive lever, which automatically stops and records the speed at the time of the fall. All testing was conducted during the animal's light cycle. All mice were tested consecutively four times and repeat measurements of speed at the time of animal falling were averaged.

Memory tests were performed using the passive avoidance test during the light phase of a standard 12 h-light and 12 h-dark cycle. The testing apparatus consists of a larger white compartment and a smaller black compartment connected by an automatic sliding door (Panlab, Harvard Apparatus, Holliston, Massachusetts, US). During the training trial, mice were placed in the white compartment facing away from the sliding door and the door was automatically opened 30 s later. The time taken to enter the black compartment was recorded. Once the mouse crossed into the black compartment, the sliding door automatically closed, and an electric foot shock was applied after a 5 s delay (0.3 mA for 5 s). Mice were removed and returned to their home cage after the shock was delivered. Testing was performed 24, 48, and 72 h after the foot shock was applied by placing mice back into the white compartment and after 30 s the sliding door was opened automatically. The latency to enter the black compartment was recorded. Once mice entered the black compartment the sliding door automatically closed, but no shock was delivered. All the measurement sequences were fully randomized to avoid any delay or any possible time-dependent artifacts.

### Microbiome Analysis

Microbiome analysis was performed on 96 fecal samples as reported previously (Snijders et al., 2016). Briefly, genomic DNA was extracted from 0.25 g of the homogenized fecal samples using the PowerSoil® DNA Isolation Kit (http://www.mobio.com/) according to instructions. PCR amplification of the V4 region of the 16S rRNA gene was carried out using the protocol developed by the Earth Microbiome Project (http://press.igsb.anl.gov/earthmicrobiome/emp-standard-protocols/16s/), and described in Caporaso et al. (2012), using improved primers described by Walters et al. (2016). Briefly, amplicons were sequenced on an Illumina MiSeq using the 250 base pair, paired end reads (http://www.illumina.com/) according to vendor's instructions. VSEARCH 2.3.0 was used to join, and quality filter demultiplexed libraries, followed by de-replication and removal of singletons and chimeras with uchime-*de novo* and uchime-ref. Reads were clustered into OTUs at 97% similarity, and taxonomy was assigned using the Qiime script assign_taxonomy.py and the Greengenes database. The centroids were aligned to Greengenes with PyNast and a phylogenetic tree was constructed using FastTree2. Sequence data are available at https://osf.io/p9fk5/. Statistical analysis and visualization were performed in R using the packages Phyloseq, DESeq2, and ggplot2. Both Bray-Curtis distances and UniFrac distances were used to compare microbial communities.

### Metabolome Analysis

Metabolites were extracted from the plasma samples using the MPLEx extraction protocol (Nakayasu et al., 2016; Snijders et al., 2016). Briefly, metabolites were extracted from 50 μL of plasma with chloroform/methanol solvent mixture, and polar and non-polar fractions were combined for the analysis while denatured protein pellets left between two separated phase layers. Extracted metabolites were completely dried and chemically derivatized for gas chromatography mass spectrometry (GC-MS) analysis. All the sample vials were sequenced in randomized order and analyzed by the instrument within a day after the derivatization. All the collected MS data files were converted to netCDF format and processed using Metabolite Detector (Hiller et al., 2009). All the peaks were matched with PNNL in-house metabolomics database which has retention index and fragmented spectra of metabolites, and additionally cross-checked with NIST14 GC-MS spectral database. All the identification went through QA/QC process to avoid misidentification of metabolites or false positive and negative errors. Raw metabolomics data are also available at https://osf.io/p9fk5/. Statistical analysis on metabolome data was performed as follows. After log2 transformation, the algorithm RMD-PAV was used to identify any potential outlier biological samples (Matzke et al., 2011), and these were confirmed via Pearson correlation and principal components analysis (PCA). The data were normalized using global median centering, and then all pairs of light conditions were compared via *t*-test with a Tukey correction. Finally, we performed partial least squares discriminant analysis (PLS-DA) using leave-one-out cross-validation on the log2 normalized data, with the plsDA function from the DiscriMiner R package (https://cran.r-project.org/web/packages/DiscriMiner/index.html). In order to do this, we first removed the 9 metabolites which had missing values (allantoin, arachidic acid, L-tryptophan, Unknown 004, Unknown 034, Unknown 050, Unknown 067, Unknown 103, and Unknown 135). We began with a single model based on the three light regimens, and subsequently moved to three models: one for DD vs. LD + LL; another for LD vs. DD + LL; and a third for LL vs. LD + DD.

### Integrative Analysis

Correlation network analysis was used on combined subsets of the log2 normalized metabolite data and 16s data. The subsets included significant metabolites and taxa as well as others which are of interest from previous studies. Metabolites used for correlation analysis were: aminomalonic acid, D-glucose, erythritol, glycolic acid, L-aspartic acid, L-tryptophan, pyruvic acid, ribitol, serotonin. Taxa used for correlation analysis were: *Bacteroidales S24-7, Bacteroidales Rikenellaceae, Clostridiales Ruminococcaceae, Clostridiales Lachnospiraceae, Turicibacterales Turicibacteraceae, Bifidobacteriales Bifidobacteriaceae, Erysipelotrichales Erysipelotrichaceae*. To get to the taxa level, OTUs corresponding to these selected taxa were summed and then log2 transformed. Spearman rank correlation analysis was performed in SPSS to measure the significance of the correlation between microbes and metabolites. Cytoscape was used to generate a correlation network where microbes and metabolites are visualized as nodes and correlation as edge.

## Results and Discussion

### The Impact of Light Exposure on Mouse Motor and Memory Behavior

**Figure 2:**
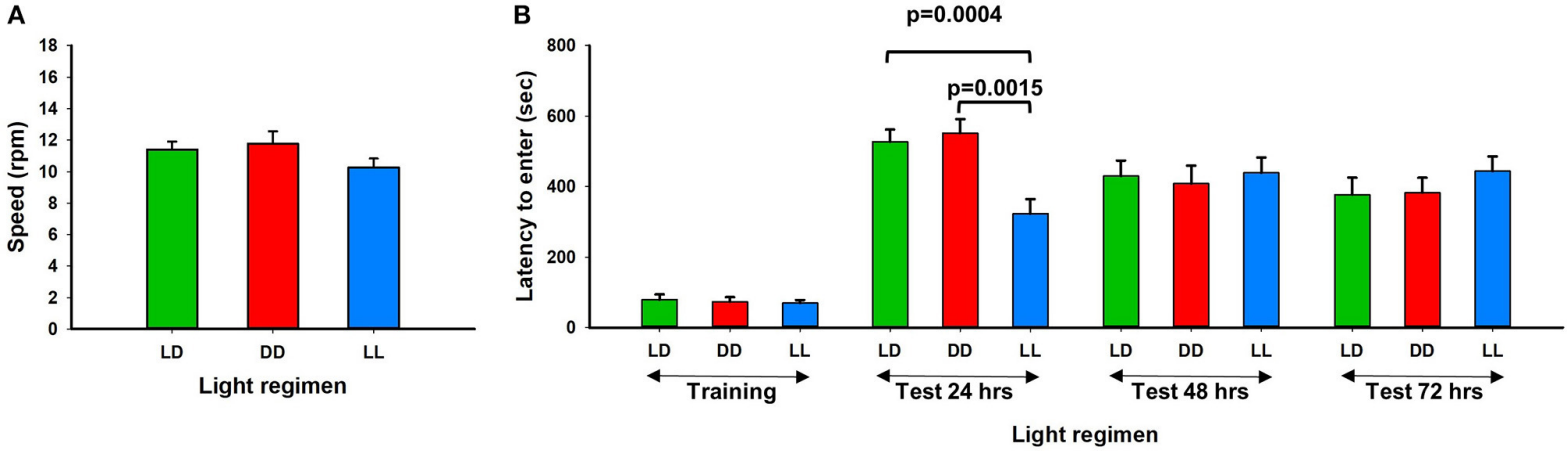
Rotarod performance and short-term memory evaluation. **(A)** Physical motor performance shown of three different light conditions. **(B)** Results of memory test from three different groups. LL mouse group showed significant memory deficiency at the first 24 h testing (Error bars are Standard Error).

Twelve weeks after exposure to continuous light (LL), continuous dark (DD), or a standard light/dark (LD) regimen, mice were acclimated to a standard light/dark regimen for 24 h and motor performance and memory potential were tested using the rotarod and passive avoidance assays. We found that motor performance was not significantly affected by the altered light-dark stress (Figure 2A). However, 24 h after foot shock, memory potential was significantly reduced in mice exposed to continuous light (Figure 2B). No difference in memory potential was observed between the different light exposure groups at 48 and 72 h after foot shock. Our results are consistent with previous reports that perturbation of the circadian rhythm in mice can influence neurobehavioral phenotypes (Reppert and Weaver, 2002; Bass and Takahashi, 2010). Richetto et al. (2018) reversed the light-dark cycles and performed behavior tests including anxiety, memory and social behavior (Richetto et al., 2018). They found that alterations in circadian and dopaminergic gene expression in mesolimbic brain structures might be involved in the different behavioral responses of mice tested in the light- vs. the dark-phase over a short-term period.

### Altered Light-Dark Stress Influences Gut Microbiome

**Figure 3:**
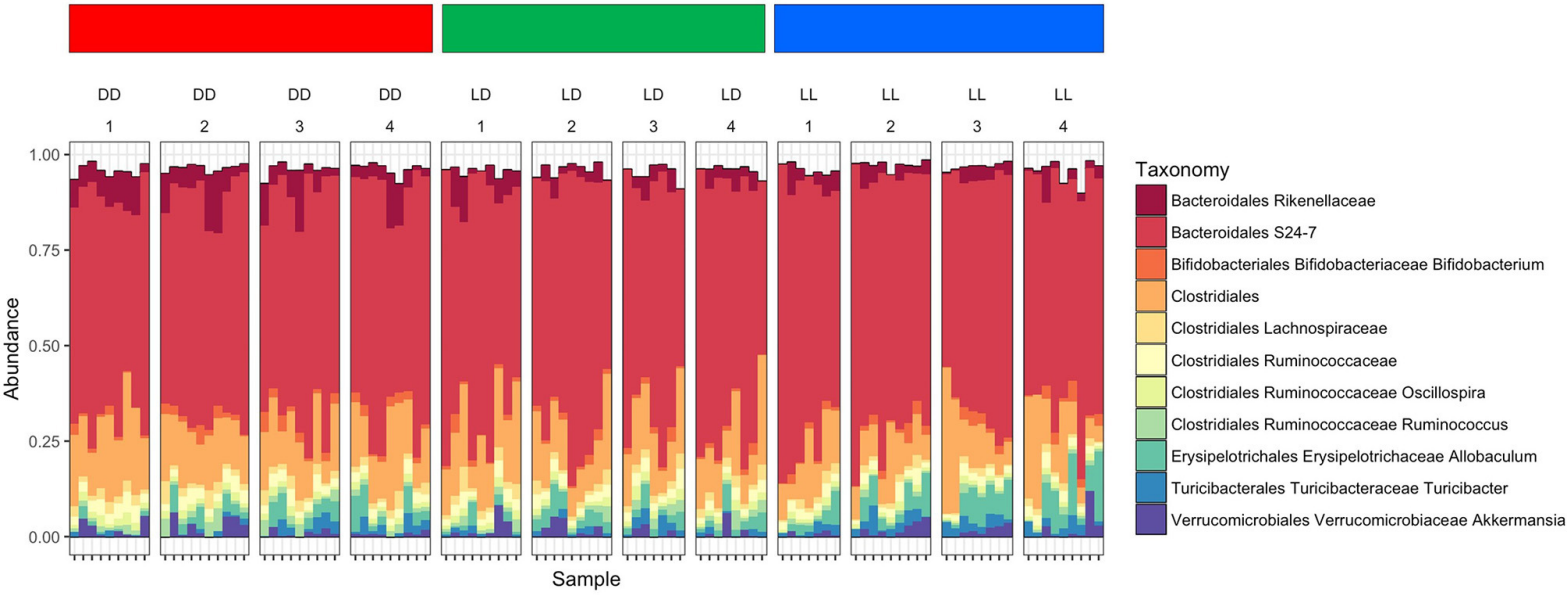
Microbiome composition under the different light conditions. Microbiome abundance based on OTUs was shown in the plot. Each column stands for the fecal sampling point over the study (1 = 0 week, 2 = 4 weeks, 3 = 8 weeks, and 4 = 12 weeks). The abundance of *Bacteroidales Rikenellaceae* in DD was higher than LD and LL, while Bacteroidales S24-7 was abundant in LL. The abundance pattern in early LL was different with DD which was showing induces stress affected microbiome composition.

**Figure 4:**
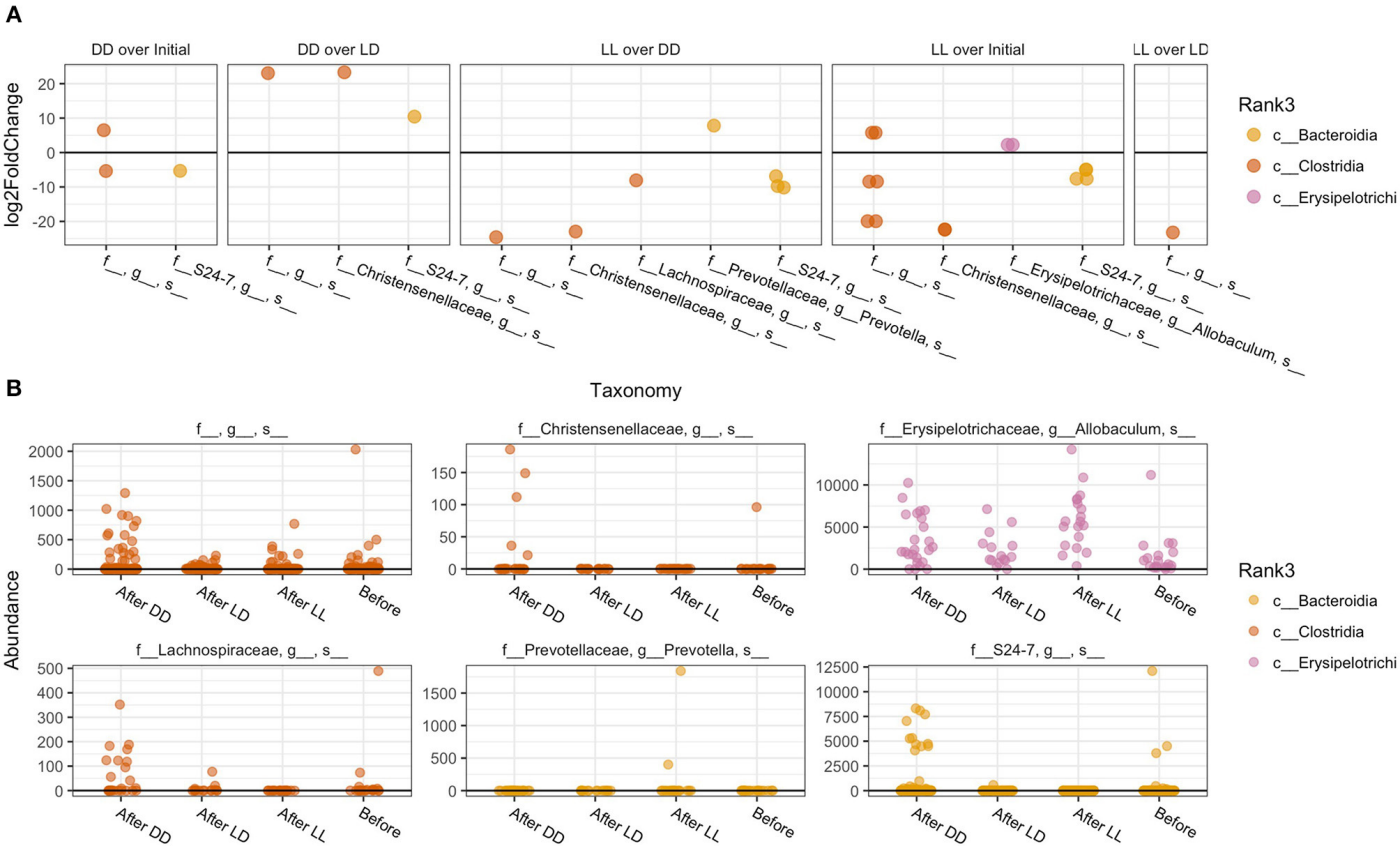
Specific taxa that significantly differed according to light regimen. **(A)** Abundance of taxa after exposure to the different light regiments and before treatment. **(B)** Differential abundance testing with DESeq2 revealed that these OTUs were significantly different between two or more of the treatment groups (adjusted *p* < 0.001).

The overall composition of the gut microbiome was not significantly changed during the different light conditions over time (Figure 3). However, the relative abundance of specific phyla was significantly different: *Bacteroidales Rikenellaceae* in DD was higher than in LD and LL, while *Bacteroidales* S24-7 was higher in LL than DD in relative abundance. Closer examination of specific taxa using differential abundance testing with DESeq2 revealed that some specific OTUs were significantly different between two or more of the treatment groups (adjusted *p* < 0.001) (Figure 4). For example, after continuous darkness several members of Clostridia were elevated, by comparison to their relative abundances prior to treatment (Figure 4A). These include an unknown bacterial strain, *Christensenellaceae*, and a strain of *Lachnospiraceae*. After the light treatment (LD or LL) the bacterium designated “S24-7” showed a lower relative abundance. Significance testing of comparisons of light treatments provided further evidence of the relative changes in specific taxa (Figure 4B).

Previous studies have shown that the differentiating taxa identified in our study responded differentially to stress and provide context for comparison to our results. Goodrich et al. (2014) previously showed that *Christensenellaceae* in the human gut is positively associated with a lean BMI index in human and addition of *Christensenella minuta* lowered weight gain in mice (Goodrich et al., 2014). This taxa is regarded as physiologically unique as shown in additional research reported that the abundance of this species was negatively associated with fat level in humans, and reduces weight gain when transplanted into germfree mice (Rosenbaum et al., 2015). Li et al. (2017) reported abundance changes of *Lachnospiraceaea* family with the host stress or diseases, and found that this genus was significantly increased when the stress was induced. The unidentified S24-7 strain was also reported in their microbiome analysis using a water immersion restraint stress in mice, but the relative abundances of that strain did not significantly change. However, our analysis revealed that S24-7 was negatively correlated with the increase of light-dark stress.

The sleep process is known to involve circadian activity (Germain and Kupfer, 2008) and gut microbes have previously been correlated to the circadian genes of hosts (Zarrinpar et al., 2014; Wang et al., 2017). In addition, the host's biological clock runs accordance with the microbiome clock (Deaver et al., 2018; Parkar et al., 2019). Our hypothesis was that the disruption of the host circadian rhythm for long term periods would change the gut microbiome equilibrium. A study showed that in circadian rhythm related gene knock out mice (*Per1/2*^−/−^ and *ASC*^−/−^) the population and functions of the intestinal microbiota lost circadian rhythm of their biochemical activities (Thaiss et al., 2014). In another study of clock gene knockout mice, the fecal microbiota showed significant changes in the rhythmicity of total load and taxonomic abundance (Liang et al., 2015). In their studies, the absolute amount of fecal bacteria and the abundance of Bacteroidetes exhibited circadian rhythmicity, which was more pronounced in female mice. *Bacterioidetes Rikenellaceae* and S24-7 that are also members of the *Bacteroidetes phylum* were also influenced by the disruption of sleep cycle in our study.

### Darkness Stress Alters Plasma Metabolome

Subsequently, we measured the metabolite composition in plasma samples collected from a subset of the previously described mice to assess metabolic indicators of light exposure; the subset comprised 34 mice in total, 12 in the LL group, 10 in the LD group, and 12 in the DD group. We detected a total of 233 metabolites of which 96 were identified and 137 remained unknown. GC-MS data went through the QC process described above, with one outlier (P_LD_828) identified and removed from subsequent analyses (Supplement Figure 3). The log2 data were then normalized via global median centering; boxplots of the samples before and after normalization are shown in Supplement Figure 2.

The metabolites that showed differences in abundance according to *t*-tests between all pairs of light regimens are shown in Table 1 as well as Supplement Tables 1, 2. For the most part, the plasma metabolome remained stable under the different light/dark conditions, as <10% (36 out of 233) of metabolites showed statistical significances from the analysis (*p*-value threshold for significance of 0.10), while only seven of the 36 metabolites have known identifications. Even though glucose levels did not pass the statistical cut-off of 0.10 for significance, it was found to be slightly lower in DD than LD, as previously shown from mice grown under short-term continuous dark conditions (Zhang et al., 2006). We observed a decrease in pyruvate and a few other key metabolites in central metabolic pathways correlated with glucose levels. Few other metabolites significantly changed between the treatments, presumably since homeostasis is the main mechanism to avoid rapid changes of metabolic activity in blood (Rosenbaum et al., 2015). Serotonin did not pass the *p*-value cut-off of 0.10 but it still showed differences in DD compared with LD and LL (Supplement Figure 1). The level of tryptophan and aspartic acid were also significantly lower in DD, whereas glycolate levels were higher (Supplement Figure 1). A recent metabolomics study on prefrontal cortex tissue reported that glutamate, homovanillic acid, lactate, pyruvate, tryptophan, uridine, D-gluconate, N-acetyl-beta-alanine, N-acetylglutamine, orotate, succinate, and methylmalonate were higher when wakefulness was enforced in the mouse study (Bourdon et al., 2018). Our data from plasma shows that tryptophan levels were higher in LL condition. This may be physiologically related with biochemical interactions in both short-term and long-term stress.

**Table 1:** Indicators of significance for each comparison, for the metabolites that were significant in at least one of the comparisons.

	**Metabolite**	**Flag_LL_vs._LD**	**Flag_LL_vs._DD**	**Flag_LD_vs._DD**
18	Aminomalonic acid	0.84	1.11	1.32^*^
42	Erythritol	0.85^*^	0.97	1.14
49	Glycolic acid	1.29^*^	1.04	0.81^*^
54	L-aspartic acid	1.08	1.14^*^	1.06
71	L-tryptophan	1.79	2.29^*^	1.28
87	Pyruvic acid	0.78^*^	1.02	1.31^*^
88	Ribitol	1.43^*^	1.10	0.77
106	Unknown 010	0.44	1.59	3.65^*^
125	Unknown 029	1.06	2.32^*^	2.18^*^
127	Unknown 031	1.22	2.22^*^	1.81
130	Unknown 034	0.70	5.19^*^	7.42^*^
132	Unknown 036	0.85	1.20	1.41^*^
135	Unknown 039	1.15	1.66^*^	1.45
140	Unknown 044	0.84	1.11	1.32^*^
149	Unknown 053	0.89	1.29	1.45^*^
155	Unknown 059	1.38^*^	1.14	0.83
159	Unknown 063	0.95	0.75^*^	0.79
162	Unknown 066	1.03	1.45^*^	1.42
166	Unknown 070	0.84	0.79^*^	0.94
169	Unknown 073	0.93	0.42^*^	0.46
174	Unknown 078	0.96	1.23	1.29^*^
178	Unknown 082	0.59^*^	0.57^*^	0.97
180	Unknown 084	1.24	0.85	0.68^*^
182	Unknown 086	0.83	1.35	1.63^*^
184	Unknown 088	0.91	1.06	1.17^*^
186	Unknown 090	1.07	0.78^*^	0.73^*^
193	Unknown 097	0.75^*^	0.91	1.21
197	Unknown 101	1.69^*^	1.15	0.68
199	Unknown 103	0.72^*^	0.81	1.12
200	Unknown 104	1.24	0.76	0.62^*^
202	Unknown 106	1.01	0.66^*^	0.66
203	Unknown 107	0.82	0.67^*^	0.81
208	Unknown 112	0.68	0.34^*^	0.50^*^
214	Unknown 118	1.17	0.76	0.64^*^
221	Unknown 125	1.86^*^	1.14	0.62^*^
226	Unknown 130	2.44	0.51	0.21^*^

*The value indicates the fold-change*.

* *indicates p < 0.10*.

**Figure 5:**
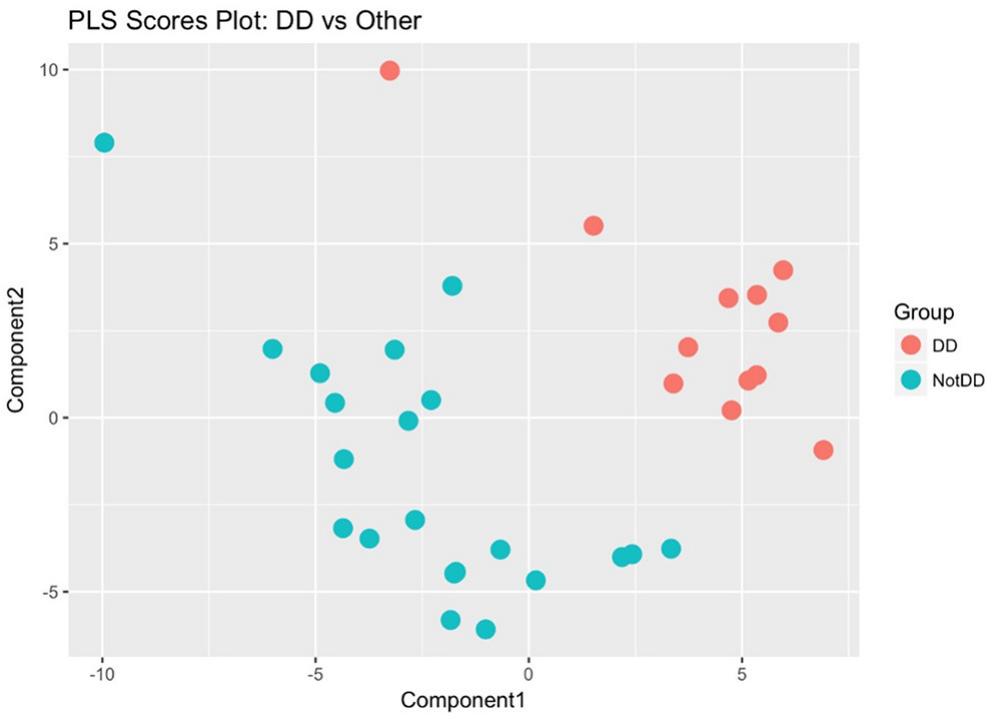
Partial Least Square regression analysis of plasma metabolome. DD samples were obviously separated against LD and LL samples. The metabolome profile of DD is distinguishable from the other conditions.

Two additional statistical analyses were performed on the global metabolome data which showed that the DD samples clustered together and separately from the LD and LL samples. The classification accuracy from the single PLS-DA model was 0.3, which is quite low and points to the possibility that two of the three groups are indistinguishable from each other. To determine whether this was the case, we re-labeled the groups and ran the following three PLS-DA models: DD vs. LD + LL; LD vs. DD + LL; LL vs. LD + DD. By comparing all of the DD samples to non-DD samples using PLS scores, we could clearly separate the metabolite profiles for the DD group from the rest of the samples (Figure 5), while other comparisons did not show such clear separations (Supplement Figures 4A,B). Probabilistic principal component analysis (PPCA) on the subset of statistically significant metabolites from the *t*-tests (Tukey-adjusted *p*-values with threshold for significance of *p* < 0.1) compared to PPCA on all of the metabolites showed noticeable improvement in terms of separation of the light regimens (Supplement Figures 5A,B). These data suggest that continuous darkness results in a different plasma composition compared to continuous light, or 12/12 light-dark cycles.

### Correlation Network Analysis Reveals Specific Relationships Between Microbiome Abundance Levels and Metabolite Levels Under Light Exposure

**Figure 6:**
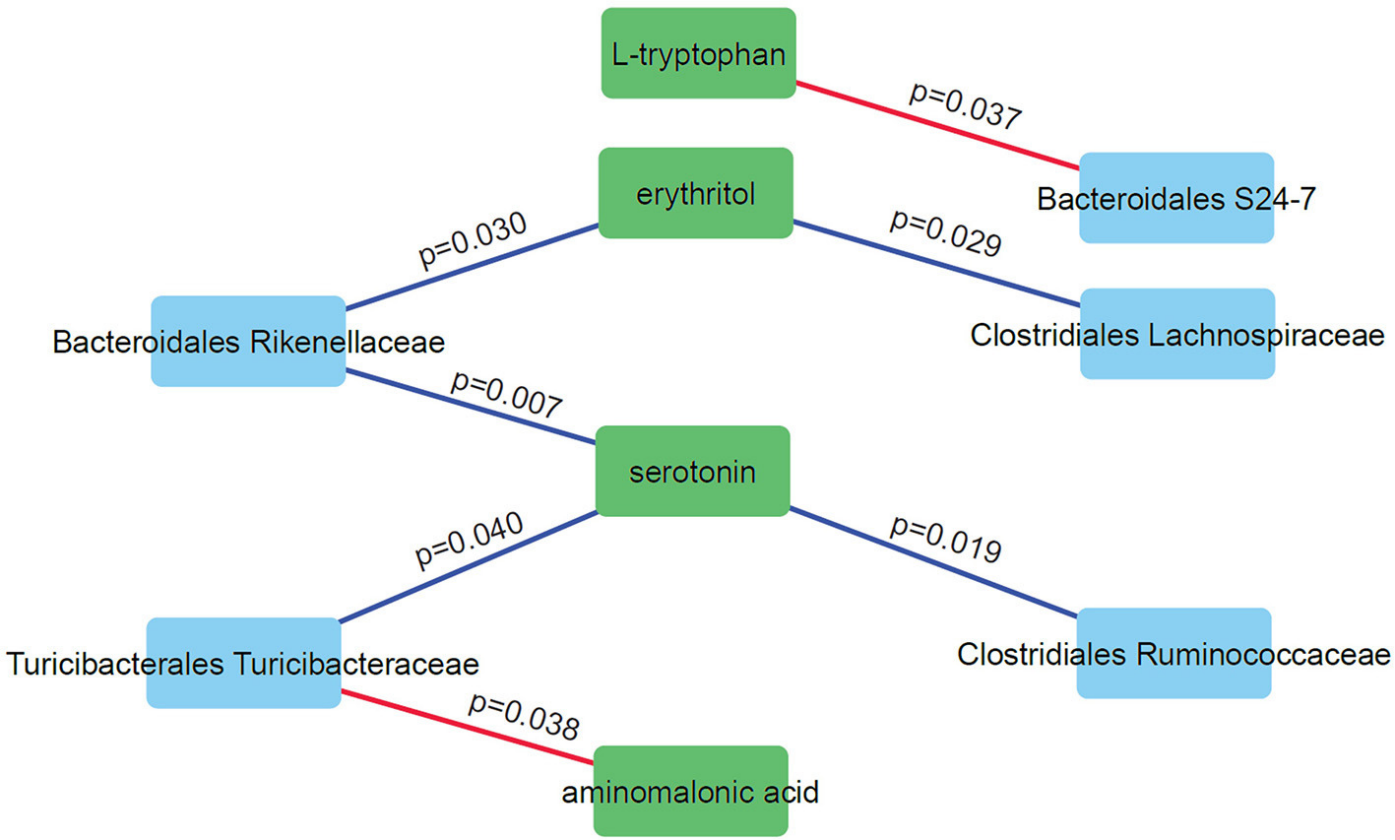
Correlation network analysis of selected microbes and metabolites. Nodes represent individual microbes (blue) or metabolites (green). Edges represent Spearman correlation between microbes and metabolites (positive correlations in blue and negative correlations in red).

We performed correlation analysis to find correlation patterns between the microbiome and metabolome using selected subsets of taxa and metabolites (Figure 6). Interestingly, we observed positive correlations between three microbial families (*Rikenellaceae, Ruminococcaceae*, and *Turicibacteraceae*) and serotonin. Serotonin is an important neurotransmitter in the human brain and has a role in regulating sleep. DD mice have significantly higher levels of serotonin compared to LD and LL (*p* = 0.028; one-way anova). Negative correlations were observed for *Bacteroidales S24-7* and L-tryptophan and between *Turicibacteraceae* and aminomalonic acid. This suggests that these particular gut microbes may influence the plasma serotonin levels under the light stressed conditions.

## Conclusion

Exposure of mice to altered light conditions had an influence on the composition of the gut microbiome and plasma metabolome. It was revealed that light stress was linked to reduced memory potential at early exposure periods. In addition, changes in the plasma metabolome were observed that correlated with changes in light/dark cycles, most likely influenced by either host and/or microbiome changes. Further research is needed to confirm the current findings of uniquely behaved individual microbes by, for example, colonizing germ-free mouse models followed by exposure to different light/dark stress conditions. These studies will help further explain the relationships between metabolites and behavior.

## Data Availability Statement

The datasets generated for this study can be found in the https://osf.io/p9fk5/.

## Ethics Statement

The animal study was reviewed and approved by Animal Welfare and Research Committee of the Lawrence Berkeley National Laboratory (Protocol File number: 271009).

## Author Contributions

Y-MK helped to design the study, perform data analysis, interpret results, and co-wrote the manuscript. AS conceived and designed the study, carried out the animal studies, performed behavior tests, collected and interpreted results, and co-wrote the manuscript. CB performed microbiome data analysis and statistics, and helped to interpret results. KS performed statistical analyses on metabolomics data. EZ prepared microbiome/metabolome samples and performed GC-MS based metabolomics analysis. SF carried out microbiome sequencing. TM interpreted results and co-wrote the manuscript. J-HM conceived and designed the study, carried out the animal studies, acquired the data, performed the data analysis, interpreted the results, and co-wrote the manuscript. JJ conceived and designed the study, interpreted the results, and co-wrote the manuscript. All authors read and approved the final manuscript.

### Conflict of Interest

The authors declare that the research was conducted in the absence of any commercial or financial relationships that could be construed as a potential conflict of interest.
