# Corrigendum: Light-Stress Influences the Composition of the Murine Gut Microbiome, Memory Function, and Plasma Metabolome

**DOI:** 10.3389/fmolb.2020.619718

**Published:** 2021-01-29

**Authors:** Young-Mo Kim, Antoine M. Snijders, Colin J. Brislawn, Kelly G. Stratton, Erika M. Zink, Sarah J. Fansler, Thomas O. Metz, Jian-Hua Mao, Janet K. Jansson

**Affiliations:** ^1^ Biological Sciences Division, Pacific Northwest National Laboratory, Richland, WA, United States; ^2^ Biological Systems and Engineering Division, Lawrence Berkeley National Laboratory, Berkeley, CA, United States; ^3^ Computing and Analytics Division, Pacific Northwest National Laboratory, Richland, WA, United States

**Keywords:** light stress, sleep cycle, gut microbiome, plasma metabolome, behavior change, memory function

In the original article, there was a mistake in the labeling three sub-headers of the Table 1, “Flag_DD_vs._LL, Flag_DD_vs.LD, and Flag_LD_vs.LL.” as published. The corrected names are “Flag_LL_vs_LD, Flag_LL_vs_DD, Flag_LD_vs_DD” appears below.

**TABLE 1:** Indicators of significance for each comparison, for the metabolites that were significant in at least one of the comparisons.

	Metabolite	Flag_LL_vs_LD	Flag_LL_vs_DD	Flag_LD_vs_DD
18	Aminomalonic acid	0.84	1.11	1.32*
42	Erythritol	0.85*	0.97	1.14
49	Glycolic acid	1.29*	1.04	0.81*
54	L-aspartic acid	1.08	1.14*	1.06
71	L-tryptophan	1.79	2.29*	1.28
87	Pyruvic acid	0.78*	1.02	1.31*
88	Ribitol	1.43*	1.10	0.77
106	Unknown 010	0.44	1.59	3.65*
125	Unknown 029	1.06	2.32*	2.18*
127	Unknown 031	1.22	2.22*	1.81
130	Unknown 034	0.70	5.19*	7.42*
132	Unknown 036	0.85	1.20	1.41*
135	Unknown 039	1.15	1.66*	1.45
140	Unknown 044	0.84	1.11	1.32*
149	Unknown 053	0.89	1.29	1.45*
155	Unknown 059	1.38*	1.14	0.83
159	Unknown 063	0.95	0.75*	0.79
162	Unknown 066	1.03	1.45*	1.42
166	Unknown 070	0.84	0.79*	0.94
169	Unknown 073	0.93	0.42*	0.46
174	Unknown 078	0.96	1.23	1.29*
178	Unknown 082	0.59*	0.57*	0.97
180	Unknown 084	1.24	0.85	0.68*
182	Unknown 086	0.83	1.35	1.63*
184	Unknown 088	0.91	1.06	1.17*
186	Unknown 090	1.07	0.78*	0.73*
193	Unknown 097	0.75*	0.91	1.21
197	Unknown 101	1.69*	1.15	0.68
199	Unknown 103	0.72*	0.81	1.12
200	Unknown 104	1.24	0.76	0.62*
202	Unknown 106	1.01	0.66*	0.66
203	Unknown 107	0.82	0.67*	0.81
208	Unknown 112	0.68	0.34*	0.50*
214	Unknown 118	1.17	0.76	0.64*
221	Unknown 125	1.86*	1.14	0.62*
226	Unknown 130	2.44	0.51	0.21*

The value indicates the fold-change.

*p < 0.10.

The authors apologize for this error and state that this does not change the scientific conclusions of the article in any way. All the raw data is still available at the Supplementary Table S2.

