# Supplementary material for: Light-Stress Influences the Composition of the Murine Gut Microbiome, Memory Function, and Plasma Metabolome
Source: Front Mol Biosci. 2019 Oct 18;6:108. doi: 10.3389/fmolb.2019.00108 (PMC6813214; doi:10.3389/fmolb.2019.00108)
Supplement: Supplementary file 3 [file Data_Sheet_1.docx]

Supplement Figures

**Supplement Figure 1.**


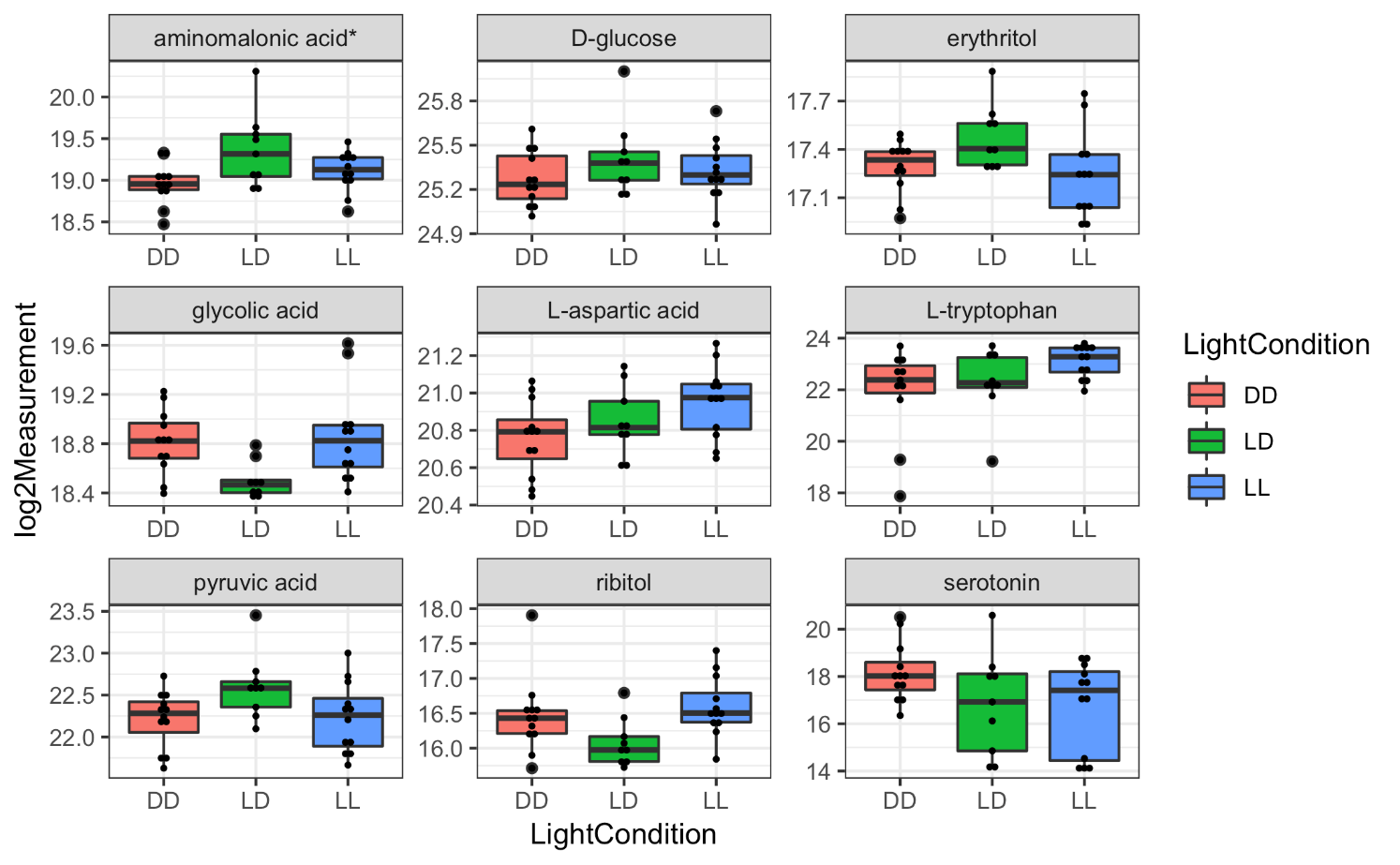


**Supplement Figure 1. Normalized log2 values for selected metabolites from the mouse metabolome analysis.**

Selected metabolites which showed patterns among the comparison of DD, LD, and LL conditions.

**Supplement Figure 2.**


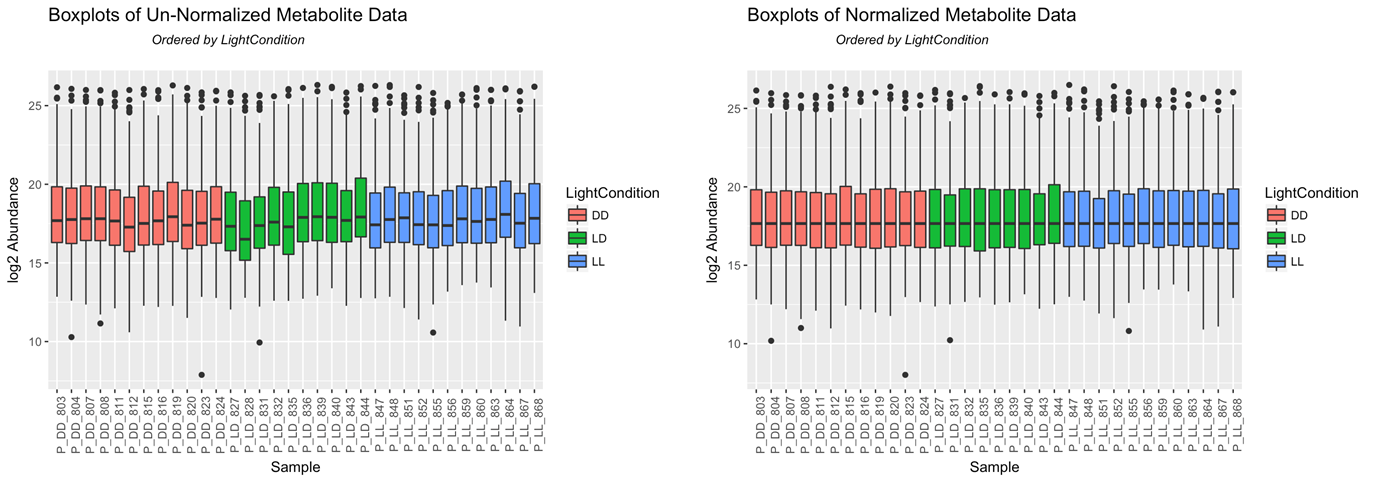


**Supplement Figure 2. Normalization of plasma metabolomics data**

A) Boxplots of un-normalized log2 metabolomics data.

B) Boxplots of normalized log2 metabolomics data.

**Supplement Figure 3.**


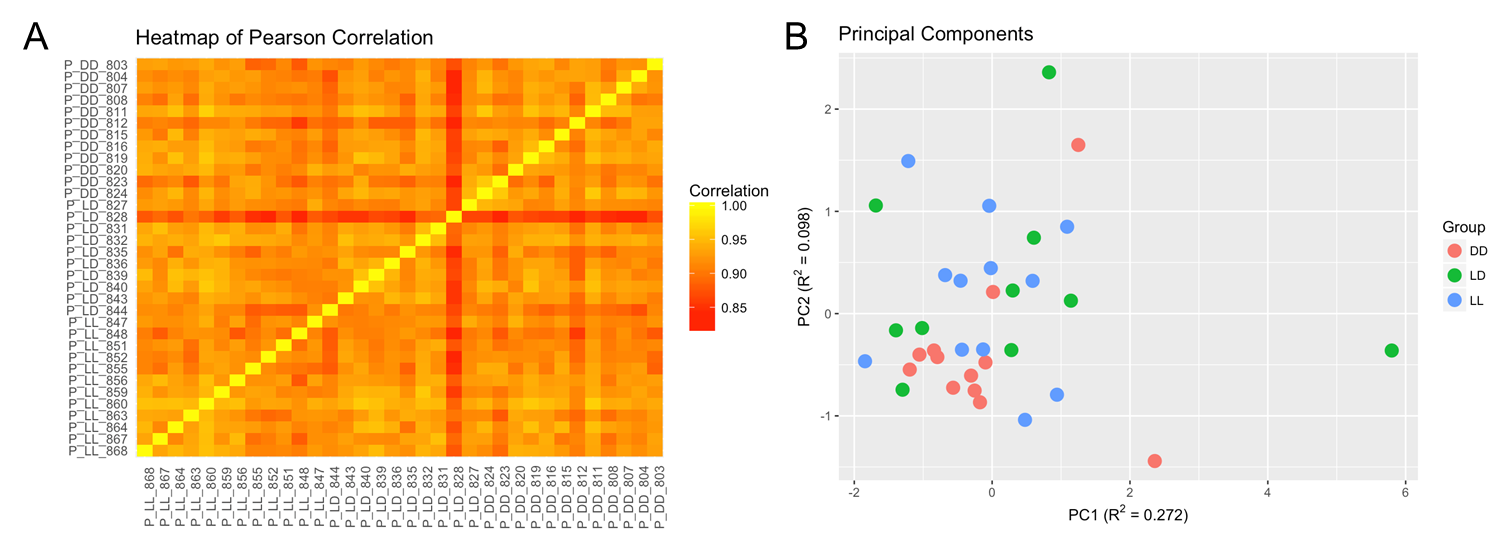


**Supplement Figure 3.**

1. Pearson Correlation Heatmap of plasma metabolome data
2. PCA plot (the coordinates of the outlying sample, P_LD_828, are (5.80 , -0.36))

**Supplement Figure 4.**


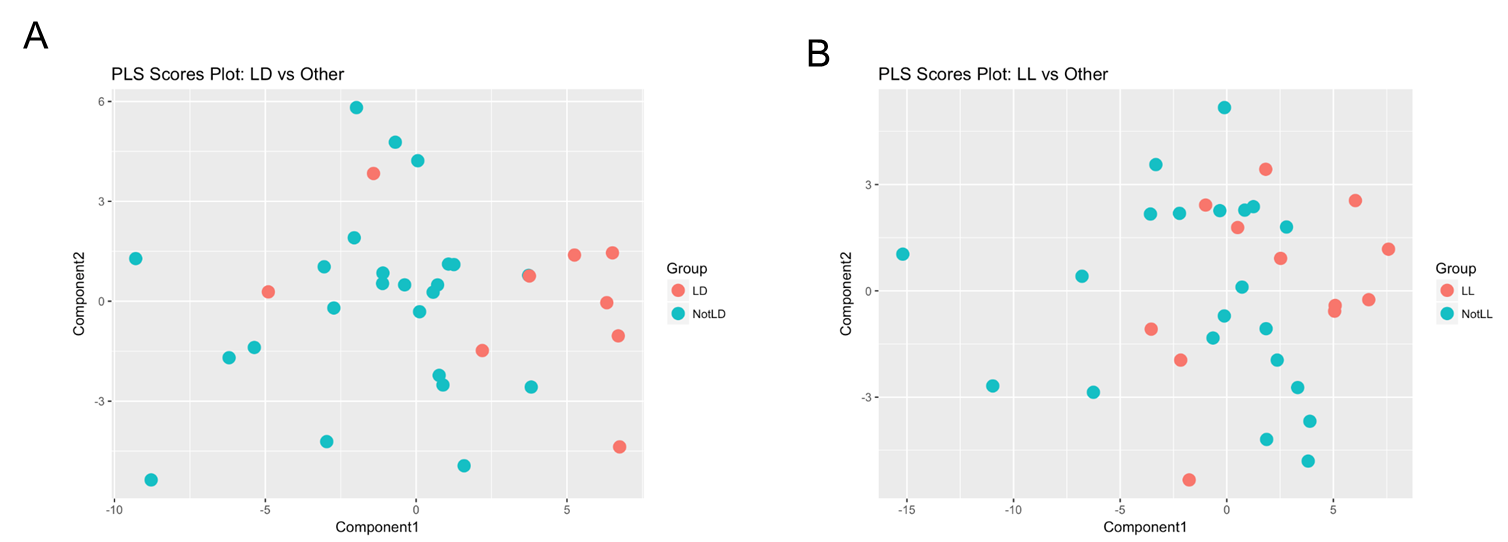


**Supplement Figure 4.**

1. PLS-DA for the comparison between LD vs other conditions (normalized log2 values)
2. PLS-DA for the comparison between LL vs the others (normalized log2 values)

**Supplement Figure 5.**


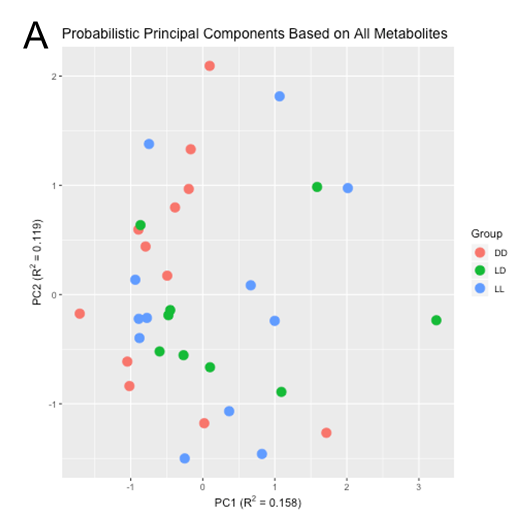

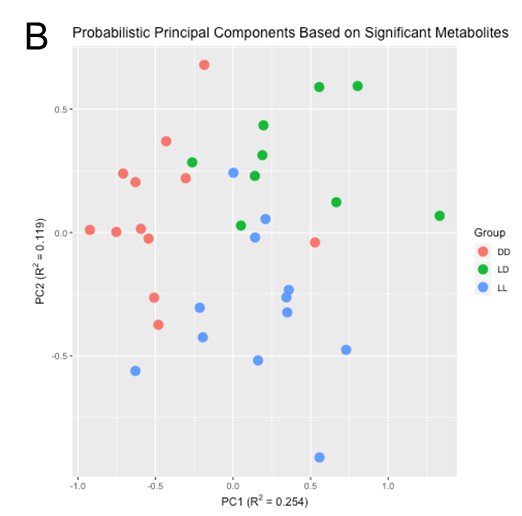


**Supplement Figure 5.**

1. Probabilistic Principal Component analysis on normalized log2 data with all the metabolites.
2. Probabilistic Principal Component analysis on log2 normalized data with all the significant metabolites from the statistical analysis (Tukey adjustment for multiple comparisons, p-value threshold for significance of 0.10).
